# Benefits of extending and adjusting the level of difficulty on computerized cognitive training for children with intellectual disabilities

**DOI:** 10.3389/fpsyg.2015.01233

**Published:** 2015-08-20

**Authors:** Jon Ottersen, Katja M. Grill

**Affiliations:** Center of Habilitation, Vestre Viken Hospital TrustDrammen, Norway

**Keywords:** intellectual disabilities, working memory, cognitive training, adaptive training, rate of failure, training amount, motivation, training intensity

## Abstract

Training on working memory (WM) improves attention and WM in children with attention-deficit hyperactivity disorder and memory impairments. However, for children with intellectual disabilities (ID), the results have been less encouraging. In this preliminary study it was hypothesized that children with ID would benefit from an extended amount of training and that the level of difficulty during training would affect the outcome. We included 21 children with mild or moderate ID aged 8–13 years. They went through between 37 and 50 training sessions with an adaptive computerized program on WM and non-verbal reasoning (NVR). The children were divided into two subgroups with different difficulty levels during training. The transfer to untrained cognitive tests was compared to the results of 22 children with ID training only 25 sessions, and to a control group. We found that the training group with the extended training program improved significantly on a block design task measuring NVR and on a WM task compared to the control group. There was also a significantly larger improvement on block design relative to the training group with the shorter training time. The children that received easier training tasks also improved significantly more on a verbal WM task compared to children with more demanding tasks. In conclusion, these preliminary data suggest that children with ID might benefit from cognitive training with longer training periods and less demanding tasks, compared to children without disabilities.

## Introduction

Working memory (WM) refers to the retention of information over a brief period of time (Klingberg, 2010). It is of major importance for a wide range of cognitive tasks and for academic achievement (Alloway and Alloway, 2010). Numerous scientific articles have concluded that cognitive functions, such as WM, can be positively influenced to higher levels by different kinds of training (Klingberg, 2010; Diamond and Lee, 2011; Hötting and Röder, 2013; Bergman-Nutley et al., 2014). Computerized WM training programs has been shown to improve WM performance in healthy groups of children and adults (Olesen et al., 2004; Jaeggi et al., 2008; Bergman-Nutley et al., 2011) and in clinical groups, such as children with attention-deficit hyperactivity disorders (Klingberg et al., 2005; Beck et al., 2010; Holmes et al., 2010; Mezzacappa and Buckner, 2010), children born preterm (Løhaugen et al., 2011) and, although sparse, children with intellectual disabilities (ID; Van der Molen et al., 2010; Söderqvist et al., 2012; Bennett et al., 2013; Delavarian et al., 2015). The training has also been shown to have far transfer effects to reduce daily life inattention (Spencer-Smith and Klingberg, 2015). Recently, a better understanding of the neural basis for cognitive development during childhood and training-induced plasticity of the brain has emerged (Klingberg, 2014), which supports the assumption that cognitive training has a positive effect.

There are different kinds of computerized programs on WM training (Klingberg, 2010). Visuospatial WM-training programs focus on retaining visuospatial information, while *n*-back training presents sequences of stimuli demanding matching of stimuli to the ones at a defined number of steps earlier in the sequence. All the programs consist of training tasks at ascending levels, and a crucial element of the training is to adjust the tasks to a challenging level of difficulty for each of the trainees all through the training in order to give optimal training progress.

Söderqvist et al. (2012) trained children aged 6–12 years with mild or moderate ID on visuospatial WM and non-verbal reasoning (NVR). The training program had been developed for a former study by Bergman-Nutley et al. (2011). The results indicated that there might be some transfer effect from training NVR to non-trained WM tasks, and Söderqvist therefore decided to utilize a program version with both WM and NVR training. The test group trained on an adaptive computerized training program ascending to more demanding levels as a result of the trainees’ right answers on the given tasks. The control group used a program with the same kind of tasks, but stayed on the easiest level throughout the entire training period. Both groups trained for 5 weeks, 5 days a week. Before training, they were tested with a battery of cognitive tests and their parents rated their behavior on a questionnaire. Their academic skills in reading, writing, number perception, and calculation was assessed by their teachers. During training, there was a large variance in progress within the test group. After training, the children were re-assessed with the same cognitive tests and their behavior was rated by their parents. One year after training, they were again tested on cognitive tests, their behavior was rated by their parents, and their academic skills were assessed at school. Comparing the results of the tests and assessments for the test group and the control group showed little difference, which indicated that the transfer effects of the adaptive training to untrained abilities were sparse. The results were compared to the study by Bergman-Nutley et al. (2011) on training 4-year-olds without special needs. It seemed like the 4-year-olds showed better transfer effects than the children in the Söderqvist et al. (2012) study, even if their cognitive capacity before training seemed to be at the same level. Söderqvist suggested that children with ID might require an alternative method of training, either by lengthening the training period or by a slower progress that allows more practice on every level.

Another important aspect that has to be taken into account for the adaptation of training is that persons with ID seem to be vulnerable in any demanding educational situation. It has been documented that children with ID report a greater frequency and intensity of fears than similar-age peers without ID (Ramirez and Kratochwill, 1997; Li and Morris, 2007). Another study showed that boys with mild levels of ID reported high levels of fear related to failure and criticism (Li et al., 2008). A high level of expectancy of failure has been a well-known phenomenon for persons with ID, probably as a result of numerous experiences of lack of success (Stancliffe et al., 2002). This expectancy can affect their motivation in such a way that task performance will be below what might be anticipated from the individual’s capabilities (Balla and Zigler, 1979; Lecavalier and Tasse, 2002). Perrig et al. (2009) stated that one of the requirements for efficient WM training for children with ID would be to ensure that the tasks are easy enough to allow success in solving the problems and to keep alive the motivation to continue training.

The authors of this article were involved in the study by Söderqvist et al. (2012). The project left a number of unanswered questions waiting to be clarified. It was therefore decided to organize an extension of the study. We chose to focus on the mechanisms involved in the training and the immediate and relatively near transfer to untrained skills and not on the longitudinal effects and the far transfer to academic and everyday skills.

Defining the aims of the study, we were inspired by Jaeggi et al. (2011) who concluded that, in addition to the amount of training, individual differences in training performance play a major role for the transfer effects. They therefore suggested that future research should pay attention to factors that moderate transfer and to find how these factors can be manipulated to make training more effective.

The main goal of the extended study was to detect possible changes in the training procedures for children with ID that could give significantly better transfer effects to non-trained tasks than what was found in the Söderqvist et al. (2012) study.

Söderqvist et al. (2012) suggested that children with ID might benefit from lengthening the training period. We had also noticed that the proportion of incorrect responses during training was relatively high in order to make the tasks sufficiently challenging for the participants. We were aware of that the participants’ experience of success and failure during training would affect their motivation on training. It would therefore be crucial to find a suitable level of difficulty on the training tasks to ensure a sufficiently high level of motivation.

For the current study the following two main hypotheses were developed:

Hypothesis 1: Children with ID will attain better transfer results on non-trained cognitive tests by extending the training period.
Hypothesis 2: The level of difficulty on the training tasks will affect training results and transfer to untrained tasks for children with ID.

The results from the present study were compared with the training group and the control group from the Söderqvist et al. (2012) study.

The participants of our group were separated into two subgroups who trained programs with different levels of difficulty.

Because of the low number of participants and its dependency on making comparisons to a former study, the present study should be considered a preliminary study.

## Materials and Methods

### Participants

E-mails were sent to every elementary school in the Oslo and Drammen regions to recruit 23 children and young adolescents. The participants all attended special education programs for children with ID and had been diagnosed with a mild or moderate mental retardation according to ICD 10 (World Health Organization, 1993). Two of the male children did not complete the training: one because of a long vacation abroad, and the other because he refused to continue training after 13 training sessions. The study included 21 participants: 10 female and 11 male, aged 8–13 years (*m* = 10.18 years, SD = 1.51).

Of the 21 participants, 10 were reported to have additional diagnoses: three with Down syndrome, two with Cerebral Palsy (with mild motor problems), two with ADHD, one with Kabuki syndrome, one with Dravet syndrome and one with William syndrome.

Ethical approvals were received from the regional ethics committee of the Norwegian south–east health region. Special information had been prepared for the children, and informed consents were obtained from the parents/caregivers and the children before participation.

Exclusion criteria were diagnosis of autism, or severe motor or sensory problems, as these were considered to affect assessments and/or training ability.

In the Söderqvist et al. (2012) study, all the children were pseudo-randomized into an intervention group or an active control group, after controlling for gender and chronological age by independent personnel. The control group trained on a non-adaptive version and the intervention group received an adaptive training program. The study had a double-blind design, with participants and the cognitive assessors unaware of group membership.

Neither the Söderqvist study nor this present study included data of parents’ socioeconomic status or educational level.

### Training Method

The participants trained on the same computerized program that was utilized in the studies by Bergman-Nutley et al. (2011) and Söderqvist et al. (2012). The program included two types of training exercises: one focused on WM training and the other on NVR training. The WM tasks are developed by Cogmed and the NVR tasks were specifically developed for the study by Bergman-Nutley et al. (2011). The level of difficulty was individually adapted by an algorithm. In this study, the number of training sessions had been extended according to the conclusions of the Söderqvist study. Because the number of training sessions had been increased from 25 to a maximum of 50, it was considered that the training could be better performed at the children’s schools. The schools were asked to implement all the 50 sessions, but if this high number would cause difficulties, 40 sessions would be sufficient. The schools were also asked to facilitate frequent training, preferably as much as five times a week. At every training session there had to be a teacher or teacher’s assistant accompanying the child.

Because of limited school resources, it was hard to recruit participants and many of the schools in this study were not able to give as much as 50 training sessions. Five children completed 37–39 training sessions, four completed 40–44, five completed 45–49, and seven completed 50. The mean number of training sessions was 44.76 (SD = 4.95). The sparse time for one-to-one teaching also resulted in that most of the participants having fewer training sessions each week, and the training was stretched out over a longer period than initially planned. The training length for our group ranged from 10 to 23 weeks.

The program had a clear structure and contained several systems of reward to keep the children motivated. During the workout, the teachers registered the children’s motivation and their way of working.

The program for NVR consisted of three alternative types of tasks. In the Classification tasks, cards with figures were to be matched on the basis of shapes, colors, and numbers. Sequential Order demanded identification of a logical progression; for instance, in position, size, or brightness. Repeated Pattern required the completion of a repeated pattern of altering shapes. Training in all the three types of tasks started at an easy level and escalated to a higher level of difficulty as a result of a given number of correct responses. Each training session started at the final level of the previous training.

The WM training consisted of seven types of tasks. Colorful figures were displayed in different settings and some of the figures made sounds and movements in a serial order. The task consisted of clicking on the figures in the same order. The number of figures to be remembered increased for each level. Each training session started at a somewhat lower level than the results at the end of the previous training session and escalated to a higher level of difficulty as a result of a given number of correct responses.

After the first 13 participants had completed their training, we changed the program. The initial program algorithm led to a task level that was considered too difficult and the high number of incorrect responses seemed to demotivate the participants.

On the NVR tasks the initial program algorithm demanded only a few correct responses on each level to escalate to the next. It seemed like the children did not acquire a real understanding of the tasks on one level before being presented to a different and more difficult set of tasks. Therefore the program used for subgroup 2 was changed to demand a higher number of correct responses to escalate to a more difficult level. The aim was to secure a better understanding and higher motivation for the participants.

On the WM tasks the number of right responses demanded to escalate was not changed, but subgroup 2, unlike subgroup 1, started each session at a level considerably lower than the final level of the previous training. The aim was to secure a feeling of mastery and success at the beginning of each session leading to a higher motivation to solve the more challenging tasks as the level of difficulty escalates.

Except for the level of difficulty, the two program versions were identical. They consisted of the same number of training sessions and the same types of tasks.

### Assessment Methods

The participants were tested at their schools by the authors of this article, before and after training, with a battery of cognitive tests. We chose to use the tests that we considered to be most suitable from the Söderqvist et al. (2012) study, supplemented with alternative tests on the same cognitive domains. The cognitive tests had been carefully selected. They had to fulfill the requirement of having indexes sufficiently fine-grained to show even little improvements, and difficulty levels adapted to ensure a feeling of success to keep the motivation steady throughout all the tests. To create a situation of predictability, the children were shown a setup with one picture for every test, and they were promised a little gift as a reward for completing the tests in order to keep them concentrated and motivated. The same procedure was followed on the pre- and post-tests.

For assessing the near transfer domain visual WM, Odd-One-Out from Automated Working Memory Assessment (AWMA; Alloway, 2007) was chosen, and for NVR, Block Design and Matrix Reasoning from Wechsler Preschool and Primary Scale of Intelligence, WPSSI-III (Wechsler, 2004a) was chosen. The domains of far transfer were considered to be verbal short-term memory and WM, and verbal reasoning. For these domains Word Span (Thorell and Wahlstedt, 2006), Comprehension of Instructions from A Developmental NEuroPSYchological Assessment, NEPSY II (Brooks et al., 2009) and Word Reasoning from WPPSI-III were chosen. In addition, Cancelation from Wechsler Intelligence Scale for Children, WISC-IV (Wechsler, 2004b) was administered in order to see if there would be a correlation between processing speed and training outcome.

After completing the training, the teachers filled out an in-house questionnaire with eight questions on a five-point scale. The questions concerned the children’s motivation during training and the teachers’ impression of the program.

### Statistical Methods

For the statistical analyses, the SPSS 21 was utilized. To test the effect of training we performed ANOVA, comparing the differences of the means of the cognitive tests before training (T1) and after training (T2) for our groups and the training- and control groups of the Söderqvist et al. (2012) study. In order to investigate correlations between the training effect and the proportion of correct and incorrect responses during training, and the number of training sessions and training intensity, we used Pearson’s *r*. To examine possible correlations between training effects (the difference between T1 and T2 scores) and the participants’ age and T1 scores, Pearson’s *r* was used.

### Subgroups

Subgroup 1 had 13 participants, five female and eight male, aged 8–13 years (*m* = 10.03 years, SD = 1.65). Subgroup 2 had eight participants, six female and two male, aged 8.5–11.5 years (*m* = 10.42 years, SD = 1.27).

The results of the cognitive tests before training (T1; **Table 1**) showed no significant differences between the subgroups.

**Table 1:** Comparisons before training of the groups from our project (Long training) and the Söderqvist study (Short training and Control) on contribution of genders, means of age (years) and means of results on cognitive tests (T1).

		Long training			Short training (*n* = 22)	Control (*n* = 19)
		Total (*n* = 21)	Subgroup 1 (*n* = 13)	Subgroup 2 (*n* = 8)		
Gender	M (No.)	10	8	2	12	10
	F (No.)	11	5	6	10	9
Age		10.18 (1.51)	10.03 (1.65)	10.42 (1.27)	9.82 (1.62)	9.53 (1.56)
T1 Block Design		21.43 (5.37)	21.23 (5.26)	21.75 (5.90)	24.27 (4.23)	23.06 (4.32)
T1 Word Span		5.19 (2.42)	5.15 (2.79)	5.25 (1.83)	5.95 (2.18)	5.26 (2.60)
T1 Odd-One-Out Memory		8.00 (6.40)	7.62 (7.97)	8.62 (2.67)	9.59 (4.29)	9.47 (4.77)
T1 Comprehension of Instructions		14.00 (4.35)	14.08 (5.25)	13.88 (2.59)	14.70 (4.97)	13.58 (4.75)

*F, female; M, male.*

We found it justifiable to merge the two subgroups on the analyses concerning training extension because both groups had trained on programs with extended number of training sessions, the two subgroups were relatively identical on age and baseline cognitive functioning and the contribution of gender was more balanced by merging the groups.

## Results

### Comparisons of Groups

The distribution of gender, the mean age and the mean results of the cognitive tests before training (T1) of the participants of this study were compared on the same variables to the test and control group of the Söderqvist et al. (2012) study. A comparison of the groups is presented in **Table 1**.

Except for the gender distribution on the subgroups, the groups were relatively similar on these variables. There were no significant differences on the age between any of the five groups. On the T1 cognitive tests there were no significant differences, but there was a trend for difference on the T1 results on Block Design between short training and long training total (*p* = 0.060) and between short training and subgroup 1 (*p* = 0.069).

### Training Progress

As we had expected, the change of the task algorithm led to an apparent reduction in the participants’ failure rates. On the NVR tasks, the participants in subgroup 1 in total had 58.7% incorrect responses, while the participants in subgroup 2 had 39.3%. On the WM tasks, subgroup 1 had 49.8% incorrrect responses, and subgroup 2 had 38.8%.

All the participants showed an overall apparent training progress, but there were large differences regarding how much they improved. Looking at the long training group as a whole, there seemed to be a steady and stable progress within both the WM and the NVR training.

**FIGURE 1:**
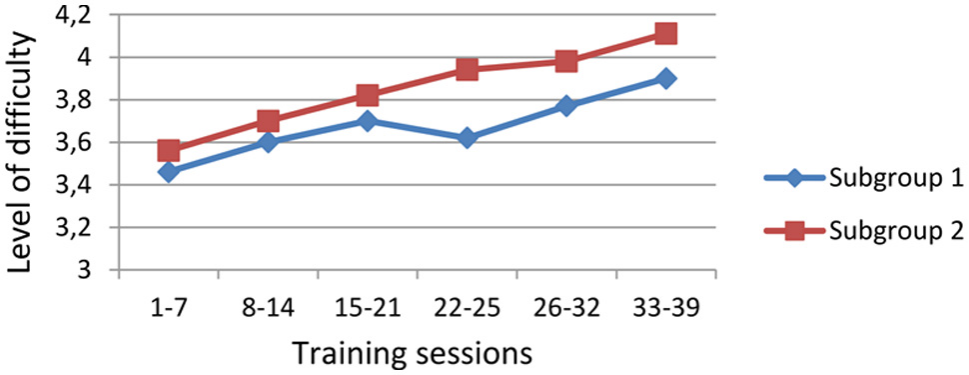
**The means of the highest levels in working memory (WM) training for the two subgroups at different stages of the training.** *Y*-axis: level of difficulty refers to the number of figures being detected and remembered. *X*-axis: training session numbers are grouped in order to contain the same variety of training tasks for each cluster.

But there was a marked difference between the subgroups in the patterns of progress during training. In the WM training the level of difficulty is escalated by increasing the number of presented figures to be remembered. Subgroup 2 started every session on lower levels, but still reached higher difficulty ratings than subgroup 1 (**Figure 1**). It seemed like subgroup 2 started each exercise at a sufficiently low level to ensure the participants’ success by easily finding the correct answer. It appeared that this adaption was beneficial, leading to higher levels of achievement.

In the NVR training the tasks are organized on subsequent levels according to the task complexity. After mastering a certain number of the tasks at one level, the participant escalates to the next level. The mean levels on the NVR training sessions 35 to 37 (where all the participants were still training) was 16.55 (SD = 7.70) for subgroup 1 and 11.65 (SD = 6.31) for subgroup 2. Subgroup 1 reached higher levels than subgroup 2, but it seemed like many of the participants had not achieved a real understanding of the nature of the task, resulting in many incorrect responses. Subgroup 2 did not reach as high levels as subgroup 1, resulting in a higher proportion of correct responses, and presumably a better understanding.

### Teachers’ Reports

In order to assess the children’s motivation and the teachers’ impression of the program, the teachers were asked to complete an in-house questionnaire using scales from 1 to 5. The mean score on motivation for the whole group was 3.78. For subgroup 1 the mean score was 3.58 and for subgroup 2 it was 4.50.

The second part of the questionnaire focused on the teachers’ judgment of the program. On the question of whether the teachers regarded the program to be too difficult, on a scale where one was “Totally disagree” and five was “Totally agree,” the mean score for the whole group was 2.56. For subgroup 1, the mean score was 3.08; for subgroup 2, it was 1.50, showing that there was a lower level of consent that the tasks were too difficult among the teachers in subgroup 2.

### Transfer to Untrained Tasks

The purpose of the cognitive tests was to detect differences in transfer of trained skills to untrained tasks between the long training groups, the short training group and the control group, as would be expected from hypothesis 1.

The mean scores and standard deviations for the groups of our study (long training) and the groups from the Söderqvist et al. (2012) study (short training and control) are presented in **Table 2**.

**Table 2:** Mean test scores and SD before training (T1) and after training (T2) for the groups of the present study (Long Training) and the Söderqvist et al. (2012) study (Short training and Control).

Tests	Long training						Short training (*n* = 22)		Control (*n* = 19)	
	
	Total (*n* = 21)		Subgroup 1 (*n* = 13)		Subgroup 2 (*n* = 8)					
					
	T1	T2	T1	T2	T1	T2	T1	T2	T1	T2
Block Design	21.43 (5.37)	24.86 (4.83)	21.23 (5.26)	24.23 (4.51)	21.75 (5.90)	25.88 (5.46)	24.27 (4.23)	25.09 (5.04)	23.06 (4.32)	22.50 (4.76)
Word Span Forward	4.00 (1.30)	4.10 (1.51)	3.85 (1.46)	3.77 (1.79)	4.25 (1.04)	4.62 (.74)	3.92 (1.94)	4.43 (1.16)	3.64 (1.62)	4.14 (1.52)
Word Span Backward	1.19 (1.57)	1.24 (1.55)	1.31 (1.70)	1.00 (1.35)	1.00 (1.41)	1.62 (1.85)	1.46 (1.32)	2.00 (1.84)	1.55 (1.77)	1.59 (1.68)
Word Span Total	5.19 (2.42)	5.33 (2.58)	5.15 (2.79)	4.77 (2.74)	5.25 (1.83)	6.25 (2.12)	5.25 (2.47)	6.43 (2.62)	5.18 (2.81)	5.73 (2.83)
Odd-One-Out Memory	8.00 (6.40)	10.81 (4.33)	7.62 (7.97)	9.77 (4.85)	8.62 (2.67)	12.50 (2.83)				
Matrix Reasoning	11.33 (5.93)	14.29 (5.07)	10.31 (6.37)	12.54 (4.88)	13.00 (5.07)	17.12 (4.22)				
Word Reasoning	9.81 (7.43)	13.62 (8.30)	9.54 (8.28)	12.54 (9.28)	10.25 (6.30)	15.38 (6.59)				
Comprehension of Instructions	14.00 (4.35)	16.33 (3.71)	14.08 (5.25)	15.69 (3.21)	13.88 (2.59)	17.38 (2.33)	14.70 (4.98)	16.20 (4.65)	14.06 (4.80)	15.12 (4.96)
Cancelation	38.33 (21.31)	45.00 (22.44)	32.77 (22.61)	40.15 (4.31)	47.38 (16.41)	52.88 (20.95)				

The difference between T1 and T2 results shows the skills gained during the training period. By comparing the differences of our group (long training total, merged by subgroup 1 and subgroup 2) and the Söderqvist et al. (2012) training group (short training), we were able to get a picture of the difference in the effect of the two training extensions. In addition, by comparing the differences between the group of long training total and the Söderqvist control group, we got an indication of the total strength of the training variable despite other variables like maturation, training effect from the pre-test, and effects of other academic training (**Table 3**).

**Table 3:** Presenting and comparing (ANOVA) test score differences from our study (long training) and the Söderqvist et al. (2012) study (short training and control).

Tests	Test score differences between T1 and T2. Means (SD)					Comparing score differences. *F* (p)		
	
	Long training			Short training (*n* = 22)	Control (*n* = 19)	Long training total (*n* = 21) vs. control (*n* = 19)	Long training total (*n* = 21) vs. short training (*n* = 22)	Subgroup 1 (*n* = 13) vs. subgroup 2 (*n* = 8)
	
	Total (*n* = 21)	Subgroup 1 (*n* = 13)	Subgroup 2 (*n* = 8)					
Block Design	3.43 (4.00)	3.00 (4.86)	4.13 (2.03)	0.82 (3.95)	0.53 (4.11)	8.57 (0.006)^∗∗^	4.64 (0.037)^∗^	0.38 (0.545)
Word Span Forward	0.10 (1.00)	-0.08 (0.86)	0.38 (1.19)	0.41 (0.85)	0.58 (1.02)	2.71 (0.137)	0.60 (0.445)	1.01 (0.325)
Word Span Backward	0.05 (1.02)	-0.31 (0.75)	0.63 (1.19)	0.18 (1.37)	0.00 (0.75)	0.12 (0.734)	1.34 (0.225)	4.92 (0.039)^∗^
Word Span Total	0.14 (1.59)	-0.38 (1.39)	1.00 (1.60)	0.59 (1.56)	0.58 (1.07)	0.76 (0.389)	0.87 (0.357)	4.39 (0.050)^∗^
Odd-One-Out Memory	2.81 (4.19)	2.15 (5.19)	3.88 (1.36)	1.86 (2.83)	0.21 (2.80)	5.20 (0.028)^∗^	0.76 (0.389)	0.83 (0.374)
Matrix Reasoning	2.95 (3.37)	2.23 (3.09)	4.13 (3.68)					1.61 (0.219)
Word Reasoning	3.81 (2.91)	3.00 (2.74)	5.13 (2.85)					2.89 (0.105)
Comprehension of Instructions	2.33 (2.41)	1.62 (2.33)	3.50 (2.20)	1.36 (2.74)	1.00 (2.79)	2.63 (0.113)	1.51 (0.226)	3.37 (0.082)
Cancellation	6.67 (14.13)	7.38 (11.28)	5.50 (18.71)					0.08 (0.775)

*^∗^p < 0.05, ^∗∗^p < 0.01.*

The Söderqvist et al. (2012) study did not find any significant differences between T1 and T2 for the short-training group and the control group. Comparing our long training group with the Söderqvist short-training group we found a significant difference on Block Design (*p* = 0.037) and in addition apparent positive differences on Odd-One-Out and Comprehension of Instructions. The comparison with the Söderqvist control group showed a significant difference on Block Design (*p* = 0.006) and also on Odd-One-Out Memory (*p* = 0.028). But on the Word Span tests the long training group showed less progress than the Söderqvist groups, mainly because of the negative differences of subgroup 1 who performed poorer on T2 than on T1.

The best over-all results we found in subgroup 2. The differences of the mean scores between T1 and T2 were larger for subgroup 2 than for subgroup 1 on all the cognitive tests except for cancelation, which was only included in the battery in order to investigate possible correlations between T1 scores and training effects. The most apparent differences between the subgroups were found on the WM tests, showing significant differences for Word Span Forward (*p* = 0.039) and Word Span Total (*p* = 0.050) and a trend of significance on Comprehension of Instructions (*p* = 0.082).

### Level of Difficulty

Hypothesis 2 focuses on the impact of the training task level of difficulty on the training effects. **Table 3** shows an overall better transfer to untrained tasks for subgroup 2, which indicates the benefit of the easier tasks. In addition, we computed the correlations between the results of the cognitive tests and the proportion of success and failures on training for the whole group. We found a significant negative correlation between the results of the Word Span test and the number and percentage of errors (incorrect responses) on the WM tasks. There was a trend of negative correlation between Word Span and the number and percentage of incorrect responses on the NVR tasks. Also, there was a trend for negative correlation between the results on the Comprehension of Instructions and the number of errors both on the WM and NVR tasks (**Table 4**).

**Table 4:** Correlation between training failure (number and percentage of errors) and the differences of the T1 and T2 test results (Pearson’s *r*).

Difference pre- and post-test		Working memory tasks		Non-verbal reasoning tasks	
			
		No. of errors	Percent errors	No. of errors	Percent errors
Word Span Total	Pearson Correlation Sig. (two-tailed) No.	-0.577^∗∗^ 0.006 21	-0.617^∗∗^ 0.003 21	-0.420 0.058 21	-0.396 0.076 21
Comprehension of Instructions	Pearson Correlation Sig. (two-tailed) No.	-0.406 0.068 21	-0.312 0.169 21	-0.379 0.091 21	-0.302 0.184 21

*^∗∗^p < 0.01.*

For the other cognitive tests, we found no significant correlations to the proportion of failure. Likewise, we found no significant correlations between any of the T1 and T2 differences on the cognitive tests and the number of correct responses or the total amount of training.

### Training Intensity

The project data also provides opportunity to investigate some additional themes, like whether training intensity affected training outcome. The number of training sessions and the number of days from training start to completion will give a picture of the intensity of the training.

There was no significant correlation between any of the results on the cognitive tests and the total amount of training. However, there was a pattern of mostly negative correlations (Block Design: *r* = 0.114; Word Span Total: *r* = -0.364; Odd-One-Out Memory: *r* = -0.248; Matrix Reasoning: *r* = -0.073; Word Reasoning: *r* = 0.000; Comprehension of Instructions: *r* = -0.340).

Likewise, there was no significant correlation between any of the results on the cognitive tests and the number of days from the start to the completion of training, measuring training intensity. (Block Design: *r* = 0.024; Word Span Total: *r* = 0.164; Odd-One-Out Memory: *r* = 0.174; Matrix Reasoning: *r* = -0.038; Word Reasoning: *r* = 0.093; Comprehension of Instructions: *r* = 0.246).

### Individual Training Benefits

It was also considered of interest to investigate whether all or just some of the participants seemed to benefit from the training. There were obvious individual differences on how many test points they improved from T1 to T2. Calculating the rank order (1–21) on the size of differences between the score results on the T1 and T2 cognitive tests (Block Design, Word Span Total, Odd-One-Out Memory, Matrix Reasoning, Word Reasoning, and Comprehension of Instructions) showed that the best mean rank order was 4.33 (SD = 2.58) and the poorest was 15.50 (SD = 6.41). For the five participants with the poorest rank order, the SD varied from 5.37 to 7.27, showing that none of them had a pervasive pattern of having the poorest improvement. This indicates that none of the participants clearly did not benefit from the training.

In order to gain information on what participants who benefited best from this particular kind of training, the transfer effects were compared to age, gender and results on the cognitive tests before training. We did not find any significant correlations between the transfer effects and the age of the participants or the T1 results. Likewise there were no significant differences comparing the mean transfer effects for the two genders.

## Discussion

### Summary of Findings

The findings showed that:

• Extended training leads to better results on non-trained tasks;
• The level of difficulty affects motivation and transfer to non-trained tasks, especially verbal WM tasks;
• Training intensity was not essential for the outcome;
• Neither age, gender nor test results before training was essential for the outcome.

### Extended Training

In the present study, we evaluated the effect of two factors: extended length of training and lower difficulty of training. On investigating hypothesis 1, results from the extended training were compared to both the training group with shorter training and the control group from the Söderqvist et al. (2012) study. We found that extended training leads to a larger improvement in non-trained tasks.

It was considered prudent to make comparisons of the results from the two studies. They had been conducted with similar procedures by the same staff. We used many of the same cognitive tests. The groups were relatively similar regarding gender, age, and cognitive abilities before training. The only apparent difference was found on Block Design. The T1 results of the short training group showed a trend of significantly better results than the long training group. However, when the long training group was compared to the Söderqvist control group, the T1 Block Design difference was not so apparent. As we found an even more promising transfer effect comparing the long training to the Söderqvist control than comparing the long training to the short training, it seems like the T1 difference on Block Design was not of major importance.

On average, our group also had a somewhat longer training period than the Söderqvist control group. Therefore, it can be argued that other kinds of learning or maturation in this extra time can positively affect the differences between T1 and T2 for our group. However, in the 14 months from the start of training to the post-tests 1 year after the completion of training, the Söderqvist control group improved the results with an average of only 0.47 points on Block Design and 1.26 points on Odd-One-Out Memory. This comparison suggests that maturation and other learning during the extra weeks of training for our group had not been of major importance for the results.

The 4-year-olds without special needs at the Bergman-Nutley et al. (2011) study gained significant transfer effects on both WM tests [Grid Task (Bergman-Nutley et al. (2010) and Odd-One-Out Memory] and on a NVR test [Leiter (Roid and Miller, 1997)]. As the participants in the current study also gained significant transfer on a WM test (Odd-One-Out Memory) and a NVR test (Block Design), it seems like children with ID can benefit from utilizing this particular training program in somewhat the same way as children on the same level of cognitive development without special needs.

We therefore find it appropriate to imply that our results suggest that extended training is beneficial to children with ID according to our first hypothesis. This assumption is supported by the fact that developmental delay is one of the main diagnostic criteria for ID, indicating a slow acquisition of new skills and a need for more repetitive trials for children with ID.

What can be considered as an optimal training length? There was also some variation as to the number of training sessions for each participant. However, those who trained in all 50 sessions did not seem to get more benefits of training and transfer than those who trained under 40 sessions. The comparisons of our results and the results from the Söderqvist groups show that the transfer effects increased by extending the number of training sessions beyond 25. Therefore, it seems that 40 training sessions would be sufficient for most of the participants. Our experience suggests that this amount of training makes it easier for the schools to fit the training into their regular program.

But children with ID constitute a heterogeneous group with obvious diversity in the patterns of cognitive functions. The differences in the relative strength and weakness of the verbal and visual skills seem to be apparent (Fletcher et al., 2004; Nuovo and Buono, 2009). Children with ID and Down syndrome generally show better skills on visual than verbal memory tasks (Van der Molen et al., 2009). This relative visual strength may explain why children with Down syndrome seem to benefit from training 25 sessions with the same WM training program from Cogmed, which was utilized in the present study (Bennett et al., 2013).

There seems to be some individual differences in cognitive profiles that need to be further investigated in order to find the best program facilitation for an optimal training efficiency.

### Levels of Difficulty

The significant transfer differences between short and long training are found on tests assessing the domains of near transfer, namely Odd-One-Out Memory on visual WM and Block Design on NVR. This is in accordance with the scientific literature that reports difficulties in transfer of skills as being one of the main characteristics of persons with ID (Beirne-Smith et al., 2006). On this basis, transfer difficulties could be considered to cause poorer transfer to distant domains with less similar elements. However, if this was the main reason, we would expect to find the same pattern in both long training subgroups. The relatively poor results on far transfer domains, like Word Span and Comprehension of Instructions on verbal WM, were found only in subgroup 1. Subgroup 2 showed a relatively even improvement on all the cognitive tests.

On the other hand, we found more promising evidence concerning the rates of failure. Subgroup 2 had a considerably lower level of difficulty than subgroup 1 (and thereby a lower percentage of incorrect responses) and better transfer to untrained tasks, indicating benefits of a lower level of difficulty. It had been considered prudent to compare the results of the two subgroups as there were no significant differences on age, training length and T1 results. There was an apparent difference on distribution of gender, but comparing the transfer effects of male and female participants showed no significant differences. There was also an apparent negative correlation between the amount of incorrect responses and the outcome results on Word Span and Comprehension of Instructions.

These results indicate support for our second hypothesis. It might be reasonable to assume that there is a connection between low motivation and/or possibly anxiety for some of the participants in our group, and the lack of improvements on the verbal WM functions. Research on WM training and motivation (Bengtsson et al., 2009; Jaeggi et al., 2014) and on WM and anxiety (Shackman et al., 2006; Visu-Petra et al., 2011; Vytal et al., 2013) can be considered to support such an assumption.

It seems like the adaptation of levels of difficulty for subgroup 1 resulted in too many incorrect responses, especially on the WM tasks. Subgroup 2, which showed the overall best transfer effects, had slightly below 40% incorrect responses on both WM and NVR training. In the first six training sessions, the error was 30.1%, and on training session 32–37 it was 40.76%. This may give an indication of an appropriate proportion of success and failure to keep motivation sufficiently high.

### Training Intensity

There was a considerable variation in the timespan from the start to the end of training and, thereby, the training intensity for our group. However, as the computations of correlation between training length and transfer effect to the cognitive tests did not show any significant values, it may be assumed that variations in training intensity did not make a great difference. This was surprising as high training intensity is recommended by the developer of the WM program (Ralph, 2014).

### Individual Training Benefits

There was no apparent pattern on the correlations between the T1 results and the transfer effects to non-trained tasks, indicating that even the participants with the lowest test scores could benefit from the training. On a preliminary basis it therefore may be considered favorable for children with ID and a cognitive developmental level approximately corresponding to an average child of 4–6 years, to perform this kind of training.

### Strengths and Limitations

Our study did not follow the ideal approach: a randomized, blinded, controlled study design, using an active control group (Klingberg, 2010). It was apparent for both the assessors, the teachers and the children that all the participants belonged to the test group, which made blinding and randomization impossible. Our results were compared to the Söderqvist control group which fulfilled the requirements of an active control group, but the accuracy of comparisons between groups from two different studies can obviously be questioned. Therefore, our results have to be considered as preliminary estimates.

The adjustments of the procedures for the cognitive testings could not be utilized for diagnostic purposes, but in this way it was possible to undertake the demanding cognitive testing of the children and thereby get credible and valid data for our project. The same procedure was also used in the Söderqvist et al. (2012) study, which made it possible to compare the results of the two projects.

The limited resources at the schools made it difficult to recruit participants. It also resulted in fewer training sessions and longer training periods than was initially planned. But these variations made it possible to do analyses of the impact of training length and intensity, which would not have been possible if the original plan had been followed.

In spite of these limitations, we consider our preliminary findings to be of importance. In a way that is different from preceding studies, we have pointed at some variables that possibly can affect the training outcome. Children with ID have strong needs for special facilitation of any educational process (Beirne-Smith et al., 2006). Therefore, it is of great importance to investigate which adaptations of cognitive training are necessary. The purpose of this study has been to focus directly on this matter.

### Ethics

The training occupies valuable time, which could have been applied for teaching other important subjects. This can only be justified if there is a compelling probability that the training has a positive effect on daily life and academic skills of the participants. At the moment, there is still a lack of evidence on long term and far transfer effects. The parents/caregivers were informed beforehand that there was uncertainty as to the effect of the training. Their motivation to consent for participation seemed to be a mixture of a hope that their children would benefit from the training and an idealistic desire to contribute to the development of new knowledge on the education of children with ID.

The majority of the teachers gave positive feedback on the program except that the tasks became too difficult for subgroup 1. The training was canceled for only one of the participants due to lack of motivation. During the testing after training, most of the children reported that even if the training sometimes had been boring, it was mainly challenging, thrilling, and enjoyable. It therefore may seem like this form of training is feasible for children with ID provided that the basic requirement of an appropriate adaptation of the degree of difficulty is taken into account. So, on a short perspective, the training seems to have been a positive experience for most of the participants. Further research is needed to answer the question of whether this kind of training should be prioritized because of the transfer effects to important functional skills.

## Conclusion

Even if there are many limitations connected to this study, the preliminary estimates show a clear tendency of better transfer results compared to the Söderqvist study, supporting our two hypotheses.

Our hope is that the results in the present study can contribute to the development of a more precise understanding of how cognitive functions can be trained to higher levels for children with ID. The results suggests that children with ID might benefit from adapted computerized training on WM and NVR in the same way as children without disabilities, provided that the training is extended and has less demanding tasks.

There are still many questions waiting for answers; therefore, there is an urgent need for more research. There is an open question of whether our results are specific only for this program. For the program that was utilized in this study, our project may be considered a pilot project, waiting for a blinded and randomized study with a higher number of participants focusing on the impact of adapting the amount of training and the level of difficulty. Furthermore there is a need for more evidence-based knowledge on long-term and far transfer effects, such as academic and everyday skills.

## Conflict of Interest Statement

The authors declare that the research was conducted in the absence of any commercial or financial relationships that could be construed as a potential conflict of interest.
